# Association between serum total cholesterol and the development of gastric cancer: A two-way two-sample Mendelian randomization study

**DOI:** 10.1097/MD.0000000000038900

**Published:** 2024-07-12

**Authors:** Peng Yan, Dong Zhao

**Affiliations:** a Department of Medical Oncology, Lixin County People’s Hospital, Bozhou, Anhui, China.

**Keywords:** causality, gastric cancer, Mendelian randomization, protective factors, total cholesterol

## Abstract

Previous epidemiologic studies have suggested a potential negative correlation between total cholesterol (TC) and Gastric cancer (GC); however, several observational studies have shown conflicting results and have failed to provide definitive evidence for a causal relationship between TC and GC. Therefore, we conducted a 2-sample bidirectional Mendelian randomization (MR) study to explore the genetic correlation and potential causal relationship between the 2 variables. We screened for single nucleotide polymorphisms (SNPs) associated with TC and GC utilizing a large-scale genome-wide association study (GWAS) public database. The causal relationship was analyzed using 5 MR analysis methods: inverse variance weighting (IVW), weighted median, MR-Egger regression, weighted mode, and simple mode. Additionally, reverse MR analysis was performed to evaluate the possibility of reverse causality. Sensitivity analyses were conducted, including heterogeneity tests, horizontal multiple validity tests, and leave-one-out tests. After meticulous screening, 79 SNPs were identified as instrumental variables (IVs). The IVW method revealed a causal relationship between TC and GC (OR = 0.844; 95% CI: 0.741–0.961; *P* = .01). Sensitivity analyses did not detect significant horizontal pleiotropy. Though heterogeneity was observed in the forward MR analysis (IVW, Q*p* = 0.0006), the results remained reliable as we utilized the IVW random-effects model as the primary analytical method. Furthermore, inverse MR analysis found no evidence of reverse causality between TC and GC, effectively ruling out the influence of GC on the reverse causality of TC. Our MR study provided evidence of a causal association between TC and GC, suggesting that TC acts as a protective factor against GC due to its negative association with the disease.

## 1. Introduction

Gastric cancer (GC) is a prevalent malignancy of the digestive system. According to the most recent global cancer data from the International Agency for Research on Cancer (IARC), newly diagnosed GC cases in 2020 constituted the 5th highest number of all newly diagnosed cancer patients, and GC-related fatalities ranked as the 3rd primary cause of cancer-related deaths, imposing a significant economic burden on families and society.^[1]^ According to cancer statistics, there were an estimated 1.09 million new cases of stomach cancer worldwide in 2020, resulting in 770,000 deaths from the disease.^[2]^

Due to the widespread use of refrigerators and advancements in living environments and dietary conditions, the global disease burden of GC has exhibited a decreasing trend in recent decades.^[3]^ However, some studies project that the absolute number of new cases of GC will continue to rise in most countries, with the incidence varying by more than eightfold between countries.^[4]^ Due to regional variations in the distribution of GC, the decreasing trend in the overall global cancer burden may obscure the true challenges in GC prevention and control in certain countries.

Deregulation of total cholesterol (TC) metabolism is also believed to be linked to cancer development.^[5]^ It has been proposed that elevated cholesterol production, acting as a precursor for various biochemical pathways (e.g., vitamin D and steroid hormones), could potentially enhance the hyperproliferation of intestinal tumor stem cells.^[6]^ Studies have indicated that the cholesterol metabolite 25-hydroxycholesterol (cholesterol 25-hydroxylase, CH25H) hinders cytosolic nibbling of cytotoxic T-lymphocytes (CTL) cells, enhances CTL activity, and impedes tumor growth.^[7]^ Studies investigating TC and tumors have revealed an association between TC levels and the development of various malignant tumors.^[8–10]^ In recent decades, numerous studies have assessed the correlation between TC levels and the incidence and mortality of GC; however, findings have been divergent, and a consensus remains elusive. Furthermore, does TC impact the development of stomach cancer? Several retrospective studies have reported no significant association between TC levels and the development of GC^[11]^; however, certain studies have demonstrated a negative correlation between TC and the development of GC^[12]^; Additionally, certain studies have indicated that elevated TC levels may elevate the risk of stomach cancer.^[13]^ At the molecular level of genes, germline pathogenic variants of 9 genes (APC, ATM, BRCA1, BRCA2, CDH1, MLH1, MSH2, MSH6, and PALB2) have been found to be associated with an increased risk of GC.^[14]^ However, there is a lack of evidence from basic research regarding the role of TC in regulating GC-related proto-oncogenes.

Mendelian randomization (MR) has been increasingly employed in recent years in epidemiological research to ascertain the causality of clinical diseases. The fundamental premise of MR studies lies in the random allocation of parental alleles to offspring during genetic inheritance, akin to the random grouping of the population. This genetic inheritance is inherent and remains fixed within the population from birth, enabling genetic variables to serve as instrumental variables (IVs) to mitigate biases introduced by reverse causality and other confounding factors. MR is now extensively utilized in genetic epidemiological studies.^[15,16]^ Results from MR analysis are considered to be less susceptible to issues such as causal inversion and confounding factors^[17]^. The present study utilized a 2-sample 2-way MR analysis to explore the causal relationship between TC and the development of GC, aiming to provide a theoretical basis for the prevention and treatment of this condition.

## 2. Information and methods

### 2.1. Study design

In this study, a 2-sample bidirectional MR study method was employed to investigate the causal relationship between TC and GC. Data on exposure and outcomes were sourced from the genome-wide association study (GWAS) database, and the analysis of the causal relationship was conducted by selecting appropriate single nucleotide polymorphisms (SNPs) as IVs using a variety of statistical methods.

### 2.2. Data sources

The data of TC and GC were obtained from the publicly available GWAS database. The data of TC were obtained from IEU OPENGWAS (https://gwas.mrcieu.ac.uk/), the sample size of the data of TC (GWASID:ieu-a-301): 187,365 people, the number of SNPs: 244,6982; GC data from GWAS Catalog (https://www.ebi.ac.uk/gwas/), GC (GWASID:GCST90018849) data sample size: 476116, number of SNPs: 24188662 (Table 1). The data utilized in this study were previously published data and therefore did not necessitate participant consent and ethics approval.

**Table 1:** Brief information on the GWAS database in the 2-sample MR study.

Project	GWASID	Database	Samplesize	Number of SNPs	Crowd
TC	ieu-a-301	IEU OpenGWAS	187365	2446982	Mixed
GC	GCST90018849	GWAS Catalog	476116	24188662	European

### 2.3. Selection of IVs

**Figure 1.**
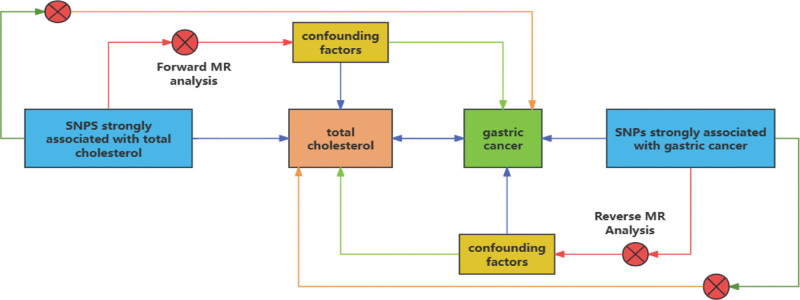
Two-sample bidirectional MR design flow chart. Forward MR analysis: TC as exposure, GC as an outcome for MR analysis. Reverse MR Analysis: GC as exposure, TC as an outcome for MR analysis. GC = gastric cancer, TC = total cholesterol, MR = Mendelian randomization.

The selection of IVs should adhere to 3 assumptions: a strong correlation between SNPs and exposure factors exists; SNPs are independent of confounding factors; SNPs can impact outcomes solely through exposure factors.^[18]^ To ascertain a significant correlation, the threshold of *P* < 10-8 was applied when TC was utilized as the exposure variable. Due to challenges in finding suitable SNPs under this constraint for screening SNPs significantly correlated with GC levels, *P* < 10-6 became the screening criterion when GC was the exposure variable. Moreover, to mitigate chain imbalance effects, a threshold of *R* < 0.001 and kb = 10000 was set. SNPs with an F-value <10 were excluded to ensure a robust correlation. SNPs for TC and GC IVs were sourced from the GWAS Catalog (https://www.ebi.ac.uk/gwas/), excluding those linked to confounders and outcomes. Subsequently, MR-PRESSO conducted an outlier test to eliminate outlier SNPs, with the remaining SNPs utilized as IVs in the study. The detailed procedural flow is depicted in Figure 1.

### 2.4. MR analysis

Various analytical methods, such as inverse variance weighting (IVW), weighted median, MR-Egger regression, weighted mode, and simple mode, were employed to explore the bidirectional causal relationship between TC and GC. The primary findings were derived from the IVW method, with the other techniques serving as supplementary and validation tools for the IVW results.^[19]^ MR results were presented as odds ratio (OR) and 95% confidence interval (CI). The Q test (Cochran Q) was applied to assess heterogeneity among IVs, with a *P* value < .05 indicating its presence. The Egger-intercept was utilized for the test of horizontal pleiotropy, focusing on the MR-Egger-intercept, where proximity to 0 suggested the absence of horizontal pleiotropy. Sensitivity analyses were conducted using the leave-one-out method, and funnel plots were utilized to assess potential bias.

### 2.5. Statistical methods

In this study, R version 4.3.1 was utilized for conducting 2-sample bidirectional MR analysis with the TwoSampleMR package and the MR-PRESSO package, with a significance level set at α = 0.05.

## 3. Results

### 3.1. Positive MR results indicate a causal association between TC levels and the development of GC

**Figure 2.**
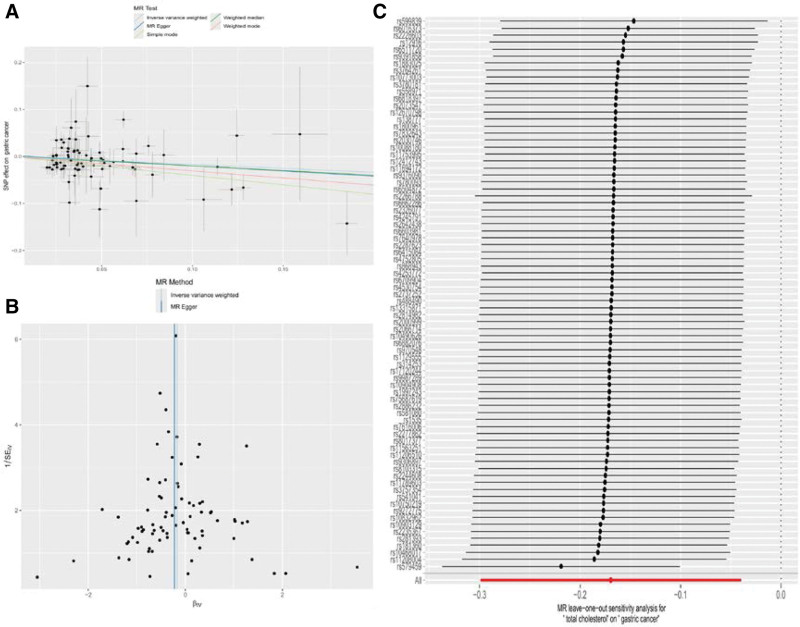
The positive relationship between TC and GC: scatter plot (A), funnel plot (B) and leave-one-out forest plot (C). Annotation: (A) Scatter plots depicting the TC-SNP associations on the x-axis and the GC-SNP associations on the y-axis were presented, accompanied by horizontal and vertical lines indicating 95% confidence intervals for each association. The downward-sloping lines from left to right indicate a negative causal relationship between the TC and GC. (B) The funnel plot showed a symmetrical distribution of effect points representing causality, indicating that they were less likely to be affected by potential bias. (C) Leave-one-out analysis was conducted to assess the causal effect of TC on GC. The black dots indicate the causal estimate of the association between a specific exposure and target after excluding each SNP sequentially. The red dots portray the overall causal estimate derived from random-effects IVW. Horizontal lines are used to illustrate 95% confidence intervals. MR = Mendelian randomization, GC = gastric cancer, SNP = single nucleotide polymorphisms, TC = total cholesterol.

**Figure 3.**
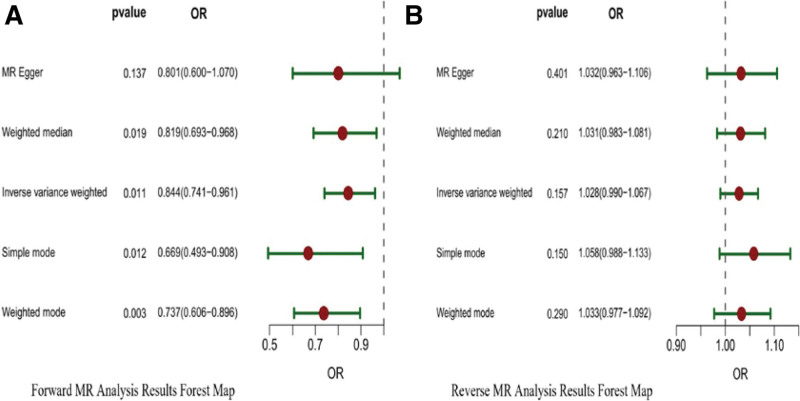
Forest plot of bidirectional relationship between TC and GC based on 5 MR analysis methods. (A) Forward MR analysis results forest map. (B) Reverse MR analysis results forest map. Horizontal axis coordinates are 95% CI ranges. GC = gastric cancer, TC = total cholesterol, MR = Mendelian randomization, OR = odds ratio.

TC was examined as an exposure variable, and GC was considered as the outcome in this analysis. SNPs significantly associated with TC were identified using a threshold of *P* < 10-8. After addressing population stratification and conducting the F-value test (retaining IVs with F-value > 10), a total of 86 preliminary SNPs were included. These 86 SNPs were cross-referenced in the GWAS Catalog website, revealing associations between rs1800562 and rs2156552 with alcohol consumption, rs3184504 with smoking, and rs4988235 and rs7412 with BMI. Furthermore, no SNPs linked to GC-associated proto-oncogenes were identified. Previous relevant clinical studies suggest that alcohol consumption, smoking, and BMI may serve as confounding factors influencing the development of GC.^[20–22]^Therefore, 5 SNPs were excluded as confounders, and 2 palindromes and incompatible sequences (rs2954029, rs378479) were removed, resulting in 79 SNPs as IVs. The IVW results indicated a negative correlation between TC and GC, which corresponded to the scatter plot (Fig. 2A). Elevated TC levels resulted in a decrease in the risk of GC by approximately 16% (OR = 0.844; 95% CI: 0.741–0.961; *P* = .01 < 0.05). All methods yielded consistent results with the IVW findings except for the MR-Egger method, and the summarized results of the 5 MR analysis methods are depicted in Figure 3A.

### 3.2. Inverse MR results of a causal association between TC and GC development

GC was studied as an exposure, with TC being the outcome. SNPs significantly associated with GC were filtered with *P* < 10-6. Subsequently, following the correction for linkage disequilibrium and the F-value test (inclusion of IVs with an F-value > 10), initially, 11 SNPs were included. Simultaneously, these 11 SNPs were cross-referenced in the GWAS Catalog website, and no SNPs related to TC were found to be confounding factors. Furthermore, no SNPs linked to GC-associated proto-oncogenes were identified. Moreover, after the exclusion of 2 palindromes and incompatible sequences (rs74511043, rs28834372), 9 SNPs were ultimately deemed suitable as IVs. The MR analysis indicated that the *P* values obtained from all 5 methods were >0.05 (refer to Table 2 and Fig. 3B), suggesting that there was no evidence supporting an inverse causal relationship between GC and the development of TC.

**Table 2:** Results of 2-way MR analysis of TC and GC.

Exposure	Outcome	MR method	Number of SNPs	OR	95%CI	*P*
TC	GC	IVW	79	0.8442	0.7414–0.9612	.0105
		MR-Egger	79	0.8010	0.5998–1.0698	.1369
		Weighted median	79	0.8186	0.6925–0.9675	.0189
		Simple mode	79	0.6693	0.4935–0.9078	.0117
		Weighted mode	79	0.7366	0.6056–0.8961	.0031
GC	TC	IVW	8	1.0275	0.9896–1.0669	.1572
		MR-Egger	8	1.0324	0.9634–1.1063	.4010
		Weighted median	8	1.0308	0.9831–1.0807	.2100
		Simple mode	8	1.0581	0.9881–1.3331	.1501
		Weighted mode	8	1.0330	0.9772–1.0920	.2899

GC = gastric cancer, TC = total cholesterol, MR = Mendelian randomization, OR = odds ratio, SNP = single nucleotide polymorphisms.

### 3.3. Sensitivity analysis

Forward MR analysis heterogeneity test (Cochran Q) showed the existence of heterogeneity in the study (Q = 125.07, *P* = .006), but a more robust random-effects IVW model was utilized to ensure the reliability of the MR analysis results. Egger-intercept was performed for the test of polytomous validity, where the intercept of MR-Egger was 0.003 close to 0 (*P* = .691), which did not support the presence of multivalence. The leave-one-out method showed little change in the overall error line after excluding each SNP (Fig. 2C). Meanwhile, the funnel plot showed a symmetrical distribution of effect points representing causality, indicating that they were less likely to be affected by potential bias (Fig. 2B). None of the reverse MR analyses revealed that the studies were heterogeneous and horizontally pleiotropic (Table 3).

**Table 3:** Sensitivity analysis.

Exposure	Outcome	Heterogeneity		Horizontalpleiotropy	
		Cochran Q	*P*	Egger-intercept	*P*
TC	GC	125.0700	.0006	0.0030	.691
GC	TC	5.2340	.6310	−0.0007	.879

GC = gastric cancer, TC = total cholesterol.

## 4. Discussion

Stomach cancer has posed a serious threat to human health and safety.^[1]^ The development of stomach cancer is influenced by a range of factors, including lifestyle, infection, and heredity. One of the most prominent factors is Helicobacter pylori infection^[23]^. There are also risk factors such as EBV infection, high salt diet, and smoking.^[24]^ Recent evidence suggests that metabolic syndrome, comprising abdominal obesity, abnormal glucose metabolism, elevated blood pressure, and dyslipidemia, is associated with GC risk.^[5]^ Previous studies have identified that the cholesterol metabolite CH25H inhibits cytosolic nibbling in CTL, enhances CTL activity, and suppresses tumor growth.^[7]^ TC levels have been identified as a protective factor against breast cancer development in breast cancer studies.^[25]^ However, the findings regarding the association between TC and GC development are still controversial. A retrospective study analyzing the preoperative lipid levels of 358 GC patients between 2001 and 2009 found that serum high-density lipoprotein (HDL)cholesterol levels were significantly correlated with cancer progression.^[11]^ The logistic regression analysis revealed that lower serum high-density lipoprotein cholesterol (HDL-C)levels were independent risk factors for deeper cancer invasion, lymph node metastasis, and advanced-stage GC.^[11]^ However, there was no significant association between other lipid markers, including triglycerides (TG), TC, low-density lipoprotein cholesterol (LDL-C), very low-density lipoprotein (VLDL), apolipoprotein A, and GC progression.^[11]^ Another prospective cohort study, which monitored 2604 patients with GC over a 14-year period, revealed that the risk of GC rose as cholesterol levels decreased. Following adjustments for confounding variables (*H pylori* infection, atrophic gastritis, family history of malignancy, smoking habits, body mass index, hemoglobin A1c, leukocyte counts, and dietary factors), this inverse correlation persisted.^[26]^ TC levels are vital for maintaining cell structure and function, as they are essential for the maintenance of biofilm structure and activity^[27]^，variations in these levels can impact a range of cellular functions, such as enzyme activity, endocytosis, and receptor function.^[28]^ The involvement of serum TC levels in the development and progression of malignant tumors is intricate, as it acts as a precursor for various biochemical pathways that synthesize crucial signaling molecules, such as vitamin D and steroid hormones, which are thought to be associated with the etiology of certain malignancies.^[29–31]^ Research has demonstrated that prolonged deficiency in serum TC can trigger the activation of nuclear factor κB, which is a crucial transcription factor that regulates immunity, inflammation, apoptosis, carcinogenesis, and other stimulating processes.^[26,28]^ Furthermore, studies have demonstrated that elevated serum cholesterol levels can increase the number of natural killer cells, leading to anti-tumor effects.^[32]^

In this study, an MR method employing SNPs as IVs was utilized, with the advantage of better avoiding the effects of various confounding factors in traditional epidemiological studies, and utilizing the publicly available GWAS database, which has a large sample size and saves time and cost, and sensitivity testing ensures result stability.^[33]^ Cancer and cardiovascular disease often share common risk factors, such as hyperlipidemia.^[34,35]^ However, Some studies have reported a positive association between cardiovascular disease and the development of lung cancer, but no causal association has been established between cardiovascular disease and the development of GC.^[36]^ The effect of cardiovascular disease as a confounding factor on the causal relationship between TC and GC was effectively eliminated, making the conclusions more reliable. While there was heterogeneity among the s used in forward MR analysis, this heterogeneity did not impact the reliability of the results thanks to the employment of the IVW random-effects model. All 5 methods in the forward MR analysis, except the MR-Egger method, yielded positive results indicating a negative association between TC and the risk of GC, thus validating the reliability of the IVW method. The insignificant results of the MR-Egger regression method could be attributed to its relatively low test efficacy. Moreover, the exclusion of reverse causality between TC levels and GC in the inverse MR analysis further strengthened the credibility of the conclusion.

However, there are limitations in this study. The GWAS data for TC were extracted from a mixed population, while the data for GC were sourced from a European population. Further research is necessary to ascertain the generalizability of the findings across different countries and regions.^[37]^ While confounding factors were accounted for, the data were not specifically analyzed according to gender and age^[37]^. Rewritten paragraph: The results of MR (MR) can only elucidate the linear relationship between omission and disease to a certain extent, but cannot be analyzed in a non-linear manner. The causal association between TC and GC needs to be verified through additional epidemiological methods.

## 5. Conclusion

In this study, the causal association between TC and GC was investigated using 2-way MR analysis as exposure factors. The findings revealed that the serum TC level acted as a protective factor against the development of GC, with a decrease in GC risk of approximately 16% in individuals with elevated serum TC levels compared to the general population. Conversely, GC did not significantly affect serum TC levels. Therefore, in the field of public health, improving the population cholesterol levels and implementing interventions for those with low cholesterol levels are crucial for preventing GC. Additionally, this research direction may inform the development of targeted drugs for GC in the future. Further investigations in vivo and ex vivo are necessary to elucidate the underlying biological mechanisms of the study results. The potential of modulating serum TC to reduce the risk of GC in clinical practice requires validation through interventional experiments.

## Acknowledgments

We are grateful to the IEU and Catalog public databases for providing the data platform, to the authors of the data, and to the authors of the R software and related packages used in the article for MR analysis, as well as to all those who have dedicated themselves to scientific research.

## Author contributions

**Conceptualization:** Dong Zhao.

**Data curation:** Peng Yan.

**Formal analysis:** Peng Yan.

**Methodology:** Peng Yan.

**Project administration:** Dong Zhao.

**Resources:** Peng Yan.

**Software:** Peng Yan.

**Supervision:** Dong Zhao.

**Validation:** Peng Yan, Dong Zhao.

**Visualization:** Peng Yan.

**Writing – original draft:** Peng Yan.

**Writing – review & editing:** Dong Zhao.
